# Secondary gliosarcoma: the clinicopathological features and the development of a patient-derived xenograft model of gliosarcoma

**DOI:** 10.1186/s12885-021-08008-y

**Published:** 2021-03-11

**Authors:** Karrie Mei-Yee Kiang, Andrian A. Chan, Gilberto Ka-Kit Leung

**Affiliations:** Division of Neurosurgery, Department of Surgery, LKS Faculty of Medicine, The University of Hong Kong, Queen Mary Hospital, 102 Pokfulam Road, Hong Kong, China

**Keywords:** Gliosarcoma, Secondary gliosarcoma, Glioblastoma multiforme, Patient-derived xenograft, Primary culture

## Abstract

**Background:**

Gliosarcoma (GSM) is a distinct and aggressive variant of glioblastoma multiforme (GBM) with worse prognosis and few treatment options. It is often managed with the same treatment modalities with temozolomide (TMZ) as in GBM. However, the therapeutic benefits on GSM from such treatment regimen is largely unknown. Patient-derived xenograft (PDX) models have been used widely to model tumor progression, and subsequently to validate biomarkers and inform potential therapeutic regimens. Here, we report for the first time the successful development of a PDX model of secondary GSM.

**Methods:**

Tissue obtained from a tumor resection revealed a secondary GSM arising from GBM. The clinical, radiological, and histopathological records of the patient were retrospectively reviewed. Samples obtained from surgery were cultured ex vivo and/or implanted subcutaneously in immunocompromised mice. Histopathological features between the primary GBM, secondary GSM, and GSM PDX are compared.

**Results:**

In explant culture, the cells displayed a spindle-shaped morphology under phase contrast microscopy, consistent with the sarcomatous component. GSM samples were subcutaneously engrafted into immunocompromised mice after single-cell suspension. Xenografts of serial passages showed enhanced growth rate with increased in vivo passage. We did not observe any histopathological differences between the secondary GSM and its serial in vivo passages of PDX tumors.

**Conclusions:**

Our PDX model for GSM retained the histopathological characteristics of the engrafted tumor from the patient. It may provide valuable information to facilitate molecular and histopathological modelling of GSM and be of significant implication in future research to establish precise cancer medicine for this highly malignant tumor.

## Introduction

Gliosarcoma (GSM) is a rare and aggressive subtype of glioblastoma multiforme (GBM), comprising up to 2.4% of GBM cases, and typically affecting the 50–70 age group with a male preponderance [1]. GSM is classified as a WHO grade IV lesion, and when compared to the usual progression of GBM, GSM carries a greater tendency of intra- and extracranial metastasis, invasion of cerebral lobes, and generally a worse prognosis. They are further classified into primary de novo GSM and secondary GSM (secondary to a recurrent or radiation-induced GBM), with a recent review noting different median survivals between the two (25 vs. 53 weeks) [1]. Clinically, GSM presents with symptoms of increased intracranial pressure and seizures, while hemiparesis, visual defects, and language deficits are infrequent [2]. On histology, GSM has two characteristic components: a glial component that expresses glial fibrillary acidic protein (GFAP) and little reticulin; and a reticulin-rich sarcomatous component lacking GFAP [3]. The currently accepted management approach for GSM is derived from the conventional treatment strategy of GBM: maximally safe surgical resection followed by chemoirradiation and adjuvant chemotherapy using temozolomide (TMZ). Due to the small volume of GSM cases limiting any form of large-scale study, there is a lack of specific treatment strategies to target this aggressive cancer.

Patient-derived xenograft (PDX) models are an essential and widely used preclinical tool for cancer medicine research, having replaced the conventional NCI-60 cell lines as a more predictive model of human cancer biology [4]. Xenografts from such conventional cell lines often fall short of clinical translation, possibly owing to these cell lines having adapted to environments beyond the natural in vivo microenvironment, and the consequent genetic changes diverging from that of natural tumor profiles in patients [5]. PDX models circumvent these limitations, with the potential for evaluating preclinical drug usage, biomarker validity, and cancer biologic studies with greater predictability of actual tumor behavior in patients [6, 7]. By collecting and directly engrafting fresh tumor tissue subcutaneously or orthotopically into immunodeficient mice, the subsequent generations of the tumor would retain the molecular, genetic, and histological heterogeneity of the sample [7]. Contemporary applications of PDX models cover different cancers such as GBM, metastatic renal cell carcinoma, colorectal cancers, lung cancers with varying degrees of success [8–14].

We report here, to the best of our knowledge, for the first time the successful development of a patient-derived xenograft model of secondary GSM with the potential for advancing personalised medicine and specific treatment for this condition.

## Materials and methods

### Tumor specimen

Fresh tumor tissue of a secondary GSM from a 59-year-old woman was collected during surgery. Formalin-fixed paraffin-embedded (FFPE) sections were obtained from the Department of Pathology, Queen Mary Hospital, Hong Kong. All specimens were obtained with informed consent from the patient. Study protocol was approved by the Institution Review Board of the University of Hong Kong/Hospital Authority Hong Kong West Cluster. Diagnosis and histological classification were confirmed by a specialist in Pathology according to the 2016 WHO brain tumor classification system of the central nervous system. (Sequencing and molecular tests listed in Table 1 were conducted by local private hospital and the pathology department respectively; experiment data is not made available in this manuscript but could be provided upon reasonable request).

**Table 1:** Clinical data, histology, and tumor marker expression of primary GBM vs. secondary GSM

Tumor	Tumor genetic and molecular profile	Histological features	Treatment
**Primary GBM**	GFAP positive Olig2 positive ATRX preserved IDH-1/IDH-2 WT 1p/19q no LOH MGMT promoter unmethylated STAG2 R216 mutation Ki-67 15–20%	Moderate neoplastic cellularity on eosinophilic fibrillary background.	Surgery + Concurrent chemoRT + Chemotherapy with TMZ Bevacizumab, carboplatin, olaparib
**Secondary GSM**	GFAP positive Olig2 positive Ki-67 ≤ 20% P53 heterogenous expression	Spindle cell morphology, distinct glial and sarcomatous component.	Surgery + bevacizumab only

*GBM* glioblastoma multiforme, *GSM* gliosarcoma, *WT* Wild type, *LOH* loss of heterozygosity, *RT* radiotherapy, *TMZ* temozolomide

### Primary culture of GSM cells

Fresh tumor tissue was washed twice with HBSS supplemented with 10% penicillin/streptomycin on ice. Tumor tissue was cut into smaller pieces (1x1x1mm) in 1 mL cold DPBS with a sterile scalpel. For cryopreservation, some tumor pieces were suspended in freezing medium (Cryostar CS10, Stemcell technologies, Canada) in a 1.5 mL cryo-tube and transferred into freezing container (Nalgene, Rochester, USA). Cryo-tubes were kept at − 80 °C overnight and transferred into liquid nitrogen tank for long term storage. To establish a primary culture cell line from the tumor explants, small tumor pieces were plated into 100 μg/mL Poly-D-Lysin (PDL, Sigma-Aldrich, St. Louis, MO, USA) coated coverslips in 4-well plate, supplemented with growth medium. Ex-vivo explant cultures were washed with PBS every 2 days and supplemented with gradually increased concentration of fetal bovine serum (FBS), from 2 to 10% in 2 weeks, to prevent outgrowth of fibroblast. Primary cells were cultured in complete growth medium of DMEM with L-glutamate (#1195–065, Gibco, Invitrogen, Carlsbad, CA, USA), supplemented with N2-supplement (1X), B27 supplement (1X), epidermal growth factor (EGF, 10 ng/mL) and basic fibroblast growth factor (bFGF, 10 ng/mL), 1% penicillin/streptomycin, and 2% FBS (all from Invitrogen). Cell cultures were maintained at 37 °C in a humidified incubator with air/CO_2_ (95:5, v/v) atmosphere.

### In vivo PDX tumor model

To develop the PDX model, immunocompromised NOD-SCID mice were used. Mice were purchased from the Laboratory Animal Unit of the University of Hong Kong, and all operations were performed according to guidelines approved by the Committee on the Use of Live Animal for Teaching and Research (CULATR). After mechanical dissociation, small tumor pieces suspended in DPBS were collected and digested with 50 μg/mL Collagenase-I (Gibco) and 0.05% Trypsin-EDTA solution (Gibco), for 20 min at 37 °C water bath (with gentle shaking every 5 min). Enzymatic dissociation was terminated with complete medium, followed by centrifugation at 100 g for 5 min. Supernatant was discarded and cells resuspended in complete medium. To obtain single cell suspension, cells were filtered and collected through a 70 μm cell strainer. Cells were resuspended in 50% (v/v) matrigel matrix (#354234, Corning, New York, USA) to facilitate tumor formation and finally engrafted subcutaneously into the right side of flank.

At the endpoint with tumor size of 2000mm^3^, PDX tumors were passaged sequentially in vivo without freezing. Tumor fragments (3x3x3mm) were extracted and directly engrafted into other mice subcutaneously. Tumor size was measured twice a week with calipers and tumor volume was calculated as: Volume = Length x Width^2^ / 2, whilst width denoted the shorter diameter.

### Immunohistochemical staining

Immunohistochemical staining of FFPE sections were performed on consecutive 5 μm-thick sections. Tissue sections were subjected to deparaffinisation by xylene, rehydration in serial dilutions of ethanol, followed by heat-induced antigen retrieval in 10 mM sodium citrate (pH 6.0). Endogenous peroxidase was quenched by treatment with 3% hydrogen peroxide (Merck Millipore, Burlington, MA, USA) for 30 min and non-specific protein binding was blocked with 10% normal goat serum (Dako, Glostrup, Denmark) for 1 h. Sections were incubated with primary antibodies at appropriate dilutions at 4 °C overnight in a moist chamber. After incubation, sections were washed with Tris-buffered saline (TBS) for three times, followed by incubation with horseradish peroxidase (HRP)-conjugated secondary antibodies (Dako) for 30 min. DAKO EnVision System (Dako) was used to detect signals from DAB chromogen substrate. Finally, sections were counterstained with haematoxylin (Vector Laboratories, Burlingame, CA) and mounted in DPX mounting solution (BDH Laboratory, UK). Primary antibodies of GFAP (#80788) and Vimentin (ab58462) were purchase from Cell Signaling Technology (Danvers, MA, USA) and Abcam (Cambridge, UK), respectively. Reticulin fibres in tissue sections were detected by reticulin silver staining (based on Gordon and Sweet’s method) according to manufacturer’s protocol (Merck Millipore).

## Results

### Clinical case presentation

**Fig. 1:**
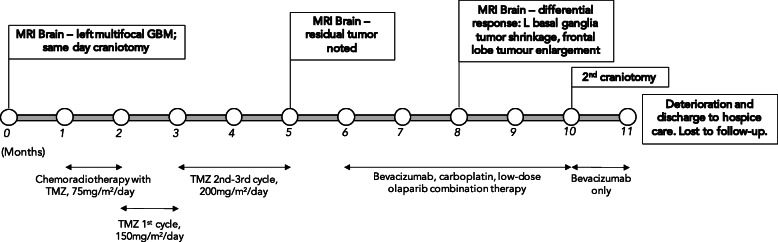
Timeline of patient diagnoses, progression, and treatment according to months since primary diagnosis

**Fig. 2:**
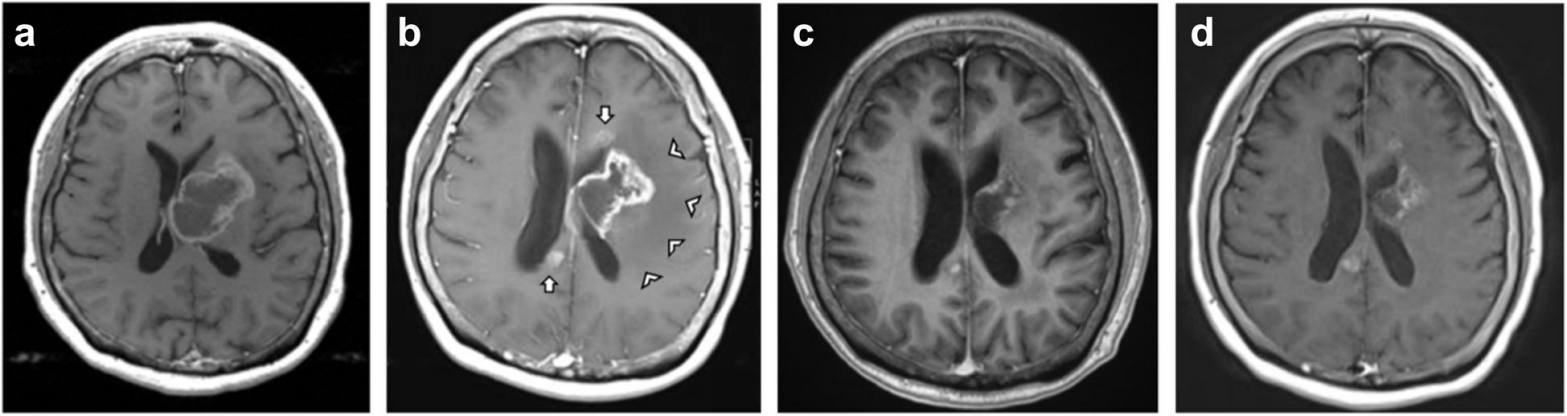
T1 axial postcontrast MRI images **a** Initial MRI showing left basal ganglia tumor with swollen left thalamus, insula cortex, and hippocampus suggesting multifocal GBM. **b** Follow up MRI 4 months after primary diagnosis showed an irregular 4.5 cm × 4.0 cm × 5.7 cm (AP x TR x CC) residual tumor. Extensive vasogenic oedema of left frontal lobe was seen extending to left insula, left hippocampal gyrus and body of corpus callosum (arrowhead). Additionally, a smaller ~ 1 cm enhancing lesions are seen, suggestive of local tumor extensions (arrowed). **c** MRI evaluation at fifth cycle of bevacizumab, carboplatin, and low-dose olaparib revealed shrinkage of left basal ganglia tumor compared to (**b**). **d** (2nd craniotomy pre-op) MRI for tumor stereotaxy and surgical planning showed an irregular 1.5 cm × 3 cm tumor mass with predominant peripheral enhancements and central necrosis at left basal ganglia, compressing on the body of the left lateral ventricle

A 59-year-old woman complained of confusion, progressive expressive dysphasia, impaired short-term memory, and gradual deterioration of cognitive function. Initial plain computed tomography (CT) scan found a 5.5 cm × 4.5 cm mass in the left frontal region involving the corona radiata, causing left lateral ventricle compression and midline shift (not shown). MRI confirmed a large necrotic tumor in the left basal ganglia and left frontal lobe (Figs. 1 and 2a). A left frontal-parietal craniotomy was performed, and the excised tumor was confirmed histologically as a primary GBM. Concurrent chemoradiotherapy with temozolomide (TMZ, 75 mg/m^2^/day) was given daily for 5 weeks. The first cycle of adjuvant TMZ (150 mg/m^2^/day) was started by the third month after primary diagnosis (Fig. 1).

Four months after the first operation, follow-up MRI showed a residual tumor mass with predominant peripheral enhancements and central necrosis at the left basal ganglia region, compressing the body of the left lateral ventricle (Fig. 2b). This lesion extended to the left frontal cortex just beneath the previous craniotomy site. Additionally, smaller ~ 1 cm enhancing lesions are seen at the left frontal lobe, left-sided genu of corpus callosum, and right sided splenium of corpus callosum, suggestive of local tumor extensions. Clinically this was correlated with a decreased Karnofsky Performance Score (KPS), deteriorated cognitive function, and dysphasia. TMZ dose was increased to 200 mg/m^2^/day for the second and third chemotherapy cycles and was completed 5 months after primary diagnosis.

Results from next-generation sequencing (NGS) of the primary tumor detected STAG2 R216 mutation, predicting genome instability and potential response to platinum-based chemotherapy and olaparib. Due to disease progression and based on the NGS results, the patient was consequently switched to bevacizumab, carboplatin, and low dose olaparib combination chemotherapy 6 months after primary diagnosis. MRI evaluation at the fifth cycle revealed shrinkage of left basal ganglia and splenium tumors (Fig. 2c), but enlargement of superior frontal lobe tumor (enlargement not shown). Further disease progression was found 10 months after primary diagnosis with post-contrast MRI showing an irregular 1.5 cm × 3 cm tumor mass at left basal ganglia with predominant peripheral enhancement and central necrosis (Fig. 2d). The patient underwent a second tumor excision, which was complicated post-operatively by repeated seizure attacks. She received hospice care thereafter. The overall survival was 47 weeks from the date of first operation. Table 1 summarises the molecular profile of the patient’s primary GBM and secondary GSM.

### Histology, genetic and molecular features of primary and secondary tumors

Immunohistochemistry of the primary GBM showed brain tissue infiltrated with neoplastic cells of moderate cellularity on an eosinophilic fibrillary background. Ki-67 proliferation index was 15–20% with prominent vascular proliferation. Neoplastic cells were positive for GFAP, Olig2 and ATRX expression, with wild-type IDH1, IDH2 and P53. There was also no loss of heterozygosity (LOH) on 1p/19q and an unmethylated MGMT promoter (Table 1). Overall, the morphology and molecular characteristics were consistent with the diagnosis of GBM.

**Fig. 3:**
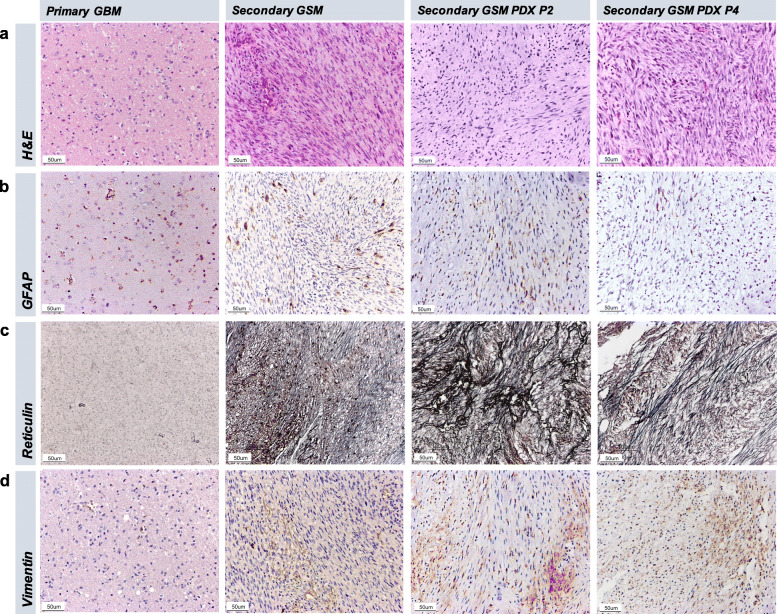
Immunohistochemical staining of FFPE sections from primary GBM, secondary GSM, PDX tumor P2 and PDX tumor P4. **a** Haematoxylin and eosin staining. **b** GFAP staining. **c** Silver staining of reticulin deposition. **d** Vimentin staining. Original magnification 200X

In the second operation, the main bulk of the excised tumor tissue was of a spindle cell morphology, composed of intersecting fascicles of spindle cells, which also invaded the brain as irregular tongues and appeared to penetrate the Virchow-Robin spaces with a perivascular growth pattern. The spindle cells possessed eosinophilic cytoplasm with indistinct borders. Moderately pleomorphic and hyperchromatic nuclei with scattered mitoses were noted (Fig. 3a). Immunohistochemistry revealed cells patchily positive for CD34, focal staining of reticulin and negative for smooth muscle actin. The glial component is positive for GFAP and Olig2, the overall histological features were consistent with the diagnosis of secondary GSM.

### Primary cell lines for GSM and GSM PDX model were successfully generated

**Fig. 4:**
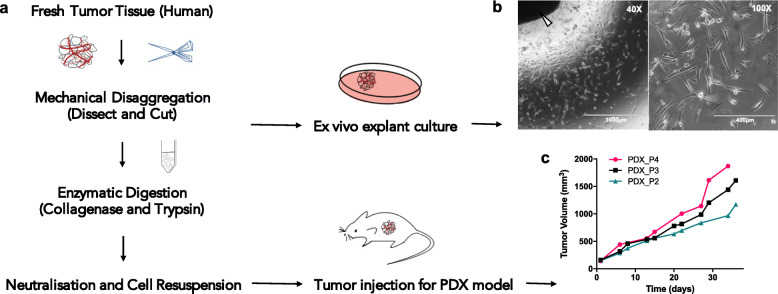
The development of GSM PDX model. **a** Flow diagram of tumor cell isolation to generate GSM primary cell line using ex vivo explant culture, and to establish animal PDX tumor model. **b** Cell morphology of explant culture under light microscope at 40X and 100X magnification. Arrowhead indicates the tumor explant tissue. **c** PDX tumor growth of sequential in vivo tumor passage P2 to P4 in NOD-SCID mice

Tumor tissue of the secondary GSM was collected immediately after the second surgery (10 months after primary diagnosis). A schematic diagram of experimental procedures is illustrated in Fig. 4a. Briefly, tumor cells were isolated by mechanical dissociation into small tumor pieces for ex vivo explant culture. Single cell suspension was obtained with further dissociation by enzymatic digestion, followed by subcutaneous tumor cell injection into immunocompromised mice. In explant culture, the cells migrated to form outgrowth at the tissue-liquid interface and displayed a spindle-shaped morphology under phase contrast microscopy, consistent with the sarcomatous component of GSM (Fig. 4b). For subcutaneous PDX tumors, tumor tissue fragments were passaged with direct transplantation without freezing. Xenografts of serial passages (P2 to P4) showed enhanced growth rate with each in vivo passage (Fig. 4c). The PDX tissues from different passages were cryopreserved for long-term storage in liquid nitrogen tank, being able to be re-implanted in vivo for future experiments.

### Histopathological characteristics of GSM are preserved in the PDX model tumors

The primary GBM and secondary GSM were distinct in terms of the additional and predominant sarcomatous component in the latter. PDX resembles the sarcomatous components seen in the patient’s tumor (GSM), both with very few gliomatous elements. We did not observe any histopathological differences between the secondary GSM and its serial in vivo passages of PDX tumors (Fig. 3a-d). GSM is characterised by its biphasic components, comprising the gliomatous (positive for GFAP, negative for Vimentin and reticulin silver staining) and sarcomatous (negative for GFAP, positive for Vimentin and reticulin silver staining) components. Here, we showed that the PDX tumors were mostly spindle-shaped cells arranged in interlacing pattern. Immunohistochemical staining patterns of both P2 and P4 PDX were also consistent with those of sarcomatoid tumor, with focal GFAP staining, reticulin and Vimentin positivity, histologically similar to those in secondary GSM. However, we observed gradual reduction of glial components in increasing passage as indicated by the reduction in GFAP expression.

## Discussion

GSM is a highly malignant and aggressive variant of GBM, with a greater capacity of intra- and extracranial metastasis [15]. However, treatment strategies specific for GSM is unavailable. It is currently managed with the same treatment modalities for GBM with tumor resection followed by chemo-irradiation and adjuvant TMZ. Current literature on GSM is also limited to mostly clinical reports. Therapeutic response of GSM to TMZ and other treatments is largely unknown, thus a more in-depth molecular and genetic characterisation of this distinct tumor entity is warranted. To date, of the four GSM PDX models established in the Mayo Clinic collection, all are identified as primary GSM [16]. In this study, we report for the first time a primary cell line and PDX animal model of a secondary GSM, not only as an invaluable tool not only for future GSM research, but also for the development of personalised cancer therapy.

Secondary GSM arising from GBM is exceedingly rare. It is more commonly seen after treatment from therapeutic irradiation of meningioma and other sarcomatous tumors. While the patient in this report received concurrent chemoradiotherapy before the diagnosis of GSM, the specific cause of GSM remained equivocal: whether it was the residual component from the heterogenous mass of the primary tumor, or a radiation-induced secondary GSM. It is noteworthy that tumor tissue resected from the second operation might have come from the residual tumor instead of developing de novo post-irradiation, when considering the tumor location within the deep brain structure where gross-total tumor resection was initially unachievable. Furthermore, tumor progression occurred with contrast-enhancing lesion and multiple masses during the third cycle of adjuvant TMZ treatment, implying that the tumor was perhaps refractory to TMZ, particularly with an unmethylated MGMT promoter. Differential response with tumor shrinkage was observed after switching to combinatorial treatment for 2 months with bevacizumab, carboplatin and olaparib. Such treatment regimen was based on the genetic test showing a STAG2 R216 mutation: it was previously reported that malignancies with STAG2 mutation is sensitive to poly-ADP ribose polymerase (PARP) inhibition (olaparib) and platinum-based chemotherapy (carboplatin) [17, 18].

The experimental procedures in developing the GSM primary culture and PDX model were conceived in the same way as those for GBM. An established GSM PDX can be used for treatment-related studies, for example, to identify the treatment response to TMZ. Here, we explicitly evaluated the growth rate and histological features at different passages. Despite most of the histopathological characteristics were preserved in the PDX tumors, tumor growth rate increased with increasing in vivo passage number. A similar phenomenon is also reported in other PDX models over multiple passages, showing a significant correlation between passage number and histopathological characteristics with greater malignant features [19]. We suggest that passage number be kept as low as possible, so as to prevent further genetic alterations and to keep the genetic profile close to the original tumor. Understanding the possible changes, and ensuring genetic heterogeneity are important determinants for accurate interpretations from data generated from PDX models.

As the technology for developing patient-derived xenograft models continue to be optimized, we foresee that this approach will bring the concept of highly personalized medicine from idealistic to reality. As previously demonstrated by Voskoglou-Nomikos and colleagues [20], these PDX models allowed for highly precise prediction of treatment response and resistance outside the patient micro-environment, with over 90% accuracy. We wish to highlight that our PDX model was derived from a single case report, largely owing to the paucity of GSM cases worldwide. The novelty of our secondary GSM PDX model is also precisely the limitation of its present-day use with regards to the higher sample size needed in identifying responders and non-responders to certain standard therapeutic regimens. Secondary GSM is a rare occurrence, but with such significantly poor outcome in patients that develop this condition, it is vital to capitalize on GSM samples whenever possible and aim to increase the development of secondary GSM PDX models. With establishing the viability of a secondary GSM PDX model for the first time, we would encourage the proactive employment and refinement of this technique to pinpoint more suitable combination therapies for each and every patient, by increasing and streamlining the capability of assessing various treatment responses.

## Conclusion

We report a new patient-derived xenograft model for gliosarcoma that preserved the histopathological characteristics of the engrafted tumor from our patient over serial passages. Current treatment strategies for gliosarcoma are equivocal, reliable biomarkers scarce, and overall prognosis very poor. This model can be useful for future preclinical studies to inform on novel molecular markers of drug response and chemoresistance, contributing to the growing library of PDX models in the refinement of personalised cancer therapy.

## Data Availability

All data generated or analysed during the current study are available from the corresponding author on reasonable request. However, the next-generation sequencing service was conducted in private, and a comprehensive list of sequencing data was not made available to the authors despite attempts to procure said data.

## Abbreviations

GBM: Glioblastoma multiforme
GSM: Gliosarcoma
TMZ: Temozolomide
PDX: Patient-derived xenograft
WHO: World health organization
CT: Computed Tomography
MRI: Magnetic resonance imaging
KPS: Karnofsky Performance Score
NGS: Next generation sequencing
H&E: Haematoxylin and eosin
RT: Radiotherapy
LOH: Loss of heterozygosity
WT: Wild type
